# From second to hundredth opinion in medicine: A global consultation platform for physicians

**DOI:** 10.1038/s41746-018-0064-y

**Published:** 2018-10-09

**Authors:** Evan D. Muse, Job G. Godino, Jessa F. Netting, James F. Alexander, Helen J. Moran, Eric J. Topol

**Affiliations:** 1 Scripps Research Translational Institute, The Scripps Research Institute, La Jolla, CA USA; 2 Division of Cardiovascular Disease, Scripps Clinic-Scripps Health, La Jolla, CA USA; 3 University of California San Diego, La Jolla, CA USA; 4 Medscape, WebMD, New York, NY USA

**Keywords:** Health care, Diagnosis

## Abstract

Serious medical diagnostic errors lead to adverse patient outcomes and increased healthcare costs. The use of virtual online consultation platforms may lead to better-informed physicians and reduce the incidence of diagnostic errors. Our aim was to assess the usage characteristics of an online, physician-to-physician, no-cost, medical consultation platform, Medscape Consult, from November 2015 through October 2017. Physicians creating original content were noted as “presenters” and those following up as “responders”. During the study period, 37,706 physician users generated a combined 117,346 presentations and responses. The physicians had an average age of 56 years and were from 171 countries on every continent. Over 90% of all presentations received responses with the median time to first response of 1.5 h. Overall, computer- and device-based medical consultation has the capacity to rapidly reach a global medical community and may play a role in the reduction of diagnostic errors.

## Introduction

Medical diagnostic errors are not infrequent and are a major cause of adverse outcomes in both the inpatient and outpatient setting.^1–3^ Outpatient diagnostic error rates have been estimated at 5% affecting >12 million individuals per annum, whereas inpatient diagnostic errors range from 6 to 7%.^2^ In fact, when using expert consultants as the ground truth for diagnosis, >20% of referral diagnoses in a recent study were vastly different compared with final diagnoses.^4^ This illustrates the importance of strong physician networks to discuss potential diagnoses and obtain second opinions on specific cases. Although informal face-to-face consultations, known more commonly as curbside consultations, are frequently inaccurate and incomplete when compared with formal professional consultations.^5,6^ However, factors relating to physician confidence, access to specialists, affordability of care, and availability of health informatics resources have all been recognized as probable barriers preventing a formalized networked approach thus leading to diagnostic errors.^3,7^

While the library stacks were once a treasure trove of valuable information to potentially crack difficult cases, the role of medical libraries and librarians has changed immensely over time.^8,9^ The growth of digital technologies has rapidly changed the way medical information is exchanged and gathered.^10^ Currently, online references and mobile applications are increasingly accessed by medical professionals than print journals.^11^ While social media and crowdsourcing have been shown to reduce costs and improve speed of information exchange, in research little has been published with regards to contemporary, virtual, peer-to-peer medical second opinion networks.^10,12–14^ While several healthcare organizations worldwide using electronic consultation (eConsult) services within their electronic health record or closed system communications to shorten the length of time it takes for patients to be evaluated by medical sub-specialists have shown improved patient and provider satisfaction, much of the published analysis of eConsult services excludes open, crowdsourced, peer-to-peer networks.^15,16^ Here, we aimed to capitalize on the global reach of the largest online community of physicians and healthcare providers (Medscape) to examine the profiles and characteristics of use within a freely available, virtual platform for crowdsourced medical consultation with over 37,000 active users.

## Results

From the launch of Medscape Consult on 10 November 2015, through 12 October 2017, there were 310,563 individual physicians who accessed the Medscape Consult platform. Of this user base, 37,706 physicians were active users and created 117,346 posts (7834 original presentations and 109,512 responses). Original presentations (patient cases) are defined here as primary content sharing either clinical cases or presenting clinical queries while responses are created by users in reply to original presentations. Examples of original presentations and responses are included as supplementary material (Supplementary Information). The majority of active users interacting on the platform created responses only, 34,046 (90.3%) users, whereas 3660 (9.7%) users created both original presentations and responses. The platform was accessible both by mobile device (Android and iOS) and personal computer. The majority of activity was created from a mobile device (67.4%) with only 32.5% of all engagements with the platform being done on a personal computer. This held true for both original presentations and responses.

**Fig. 1:**
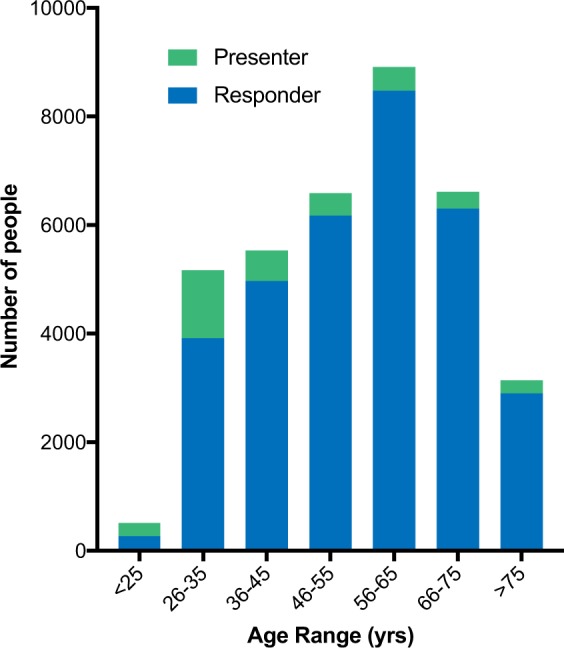
Platform activity by age group. Number of active users (presenters in green and responders in blue) by age group using the Medscape Consult platform during the study period

The mean age of all users on the platform was 54.5 (SD 15.7) years with users who created original presentations being younger, mean age 44.7 (SD 17.7) years, as compared with users who solely provided responses, mean age 55.5 (SD 15.2) years. This is reflected in Fig. 1 showing that younger users represented a larger proportion of users creating original presentations than older users.

Overall, users identified as having obtained an M.D. (75.3%), non-U.S. M.D. equivalent (7.2%), or Doctor of Osteopathic Medicine (D.O.) (3.0%), though (14.5%) of users did not specify their highest degree of training. Platform users were registered in 171 separate United Nations defined countries representing every continent. The majority of users were registered in the United States (33.7% of total users), followed by users in Europe (19.2%), Latin America (14.3%), North Africa and Western Asia (11.2%), and Central/Eastern Asia (10.8%). However, original presentation activity came from a diverse user base, with users creating original presentations registered in the United States (26.3% of users who created presentations), North Africa/Western Asia (25.3%), and Central/Eastern Asia (21.6%) representing the top three regions.

**Fig. 2:**
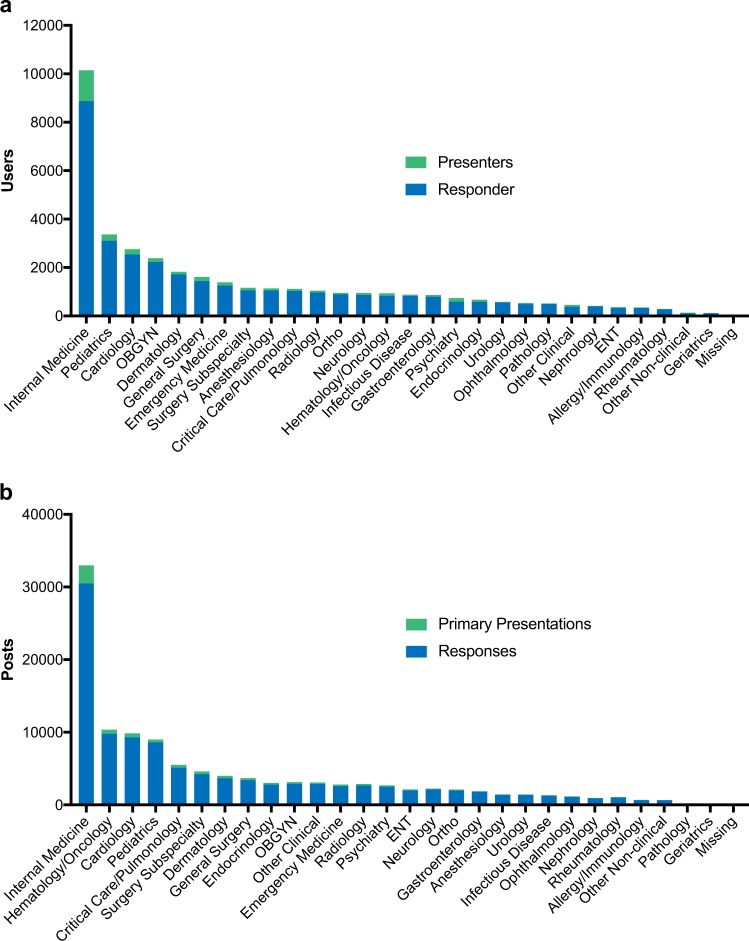
Physician activity on the platform based on medical subspecialty. **a** Number of active users (presenters in green and responders in blue by medical specialty. **b** Number of posts (presentations in green and responses in blue) made during the study period by each medical specialty

Over a quarter of the users on the platform who had either posted original presentations or made responses identified Internal Medicine (26.9%) as their primary medical specialty (Fig. 2a). Pediatrics (8.9%), Cardiology (7.3%), Obstetrics and Gynecology (6.3%), and Dermatology (4.8%) were the next most populous specialties making up the top five user groups. In terms of overall activity, users from Internal Medicine provided the most original presentations (31.5%), as well as responses (27.9%) (Fig. 2b). In general, content creation (presentations and responses) on the platform mirrored overall user percentages, with some exceptions. While Dermatology, accounting for the fifth most popular user group (4.8%), provided the second highest percentage of responses (9.0%), they were seventh (4.3%) in creation of original presentations. Alternatively, Obstetrics and Gynecology was the fourth most popular user group (6.3%) on the platform but ranked 10th in providing original presentations (3.1%) though ranking 5th (4.7%) in terms of overall comments.

**Fig. 3:**
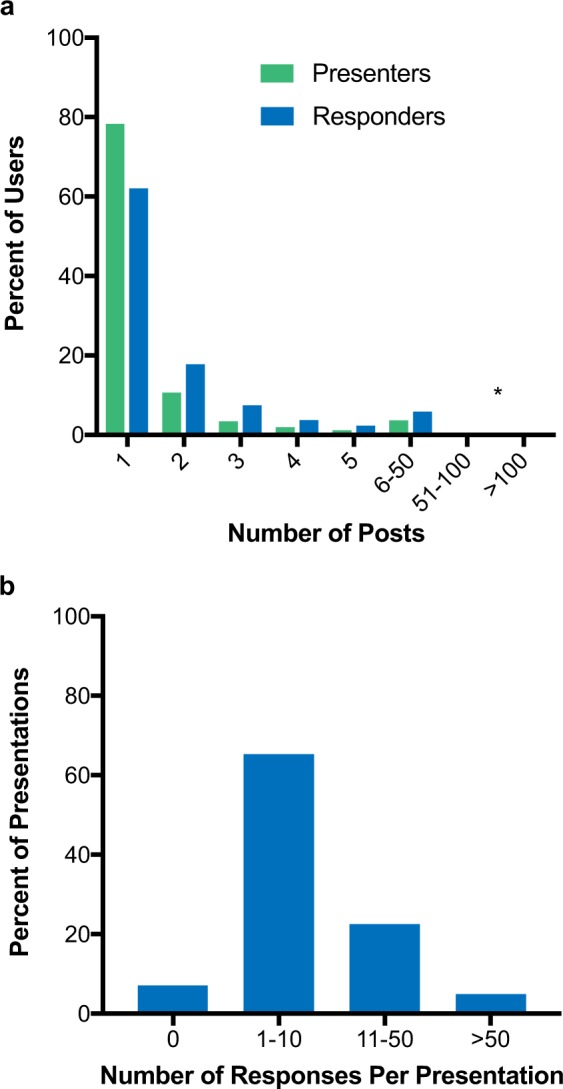
Users who engage in post activity. **a** Percent of physicians creating original content by number of posts (presenters in green and responders in blue). *0.14%, 0.05%, 0.16%, and 0.06% for presenters and responders for groups 51–100 and >100, respectively. **b** Proportion of original presentations receiving responses

Of the 7834 original presentations and 109,512 responses, the majority of users creating content did so on a single occasion with 78.2% of the users creating a single original presentation and 62.0% creating a single response (Fig. 3a). However, five users created between 51 and 100 original presentations, and six users created >100 original presentations each, with 212 posts by a single user being the highest per individual. While users creating responses were more likely than users creating presentations to do so more than once, the median for each group was 1. And, like users creating original presentations, there were several “super users”, with 16 individuals creating between 51 and 100 responses and 19 users who created >100 responses, with the highest being 1748 responses by a single user. The 3660 users (9.7% of total platform users) creating original presentations also created 27.4% of the total responses on the platform during the study period. Additionally, of the entire physician population accessing the platform (310,563 physicians) during the study period, two-thirds returned to the site more than once.

Most original presentations generated interest by responders with 65.2% of original presentations receiving between 1 and 10 responses (Fig. 3b). Although 7.2% of the original presentations received no responses from other users. The median number of responses per original presentation was 5.4 and the maximum number of responses created in reply to a single presentation was 880. For each presentation initiated on the platform that received a response, the median time to first response was 1.5 h. The first response to nearly a quarter of these original presentations (22.6%) occurred within 30 min and within at least 15 h for 89.2%.

**Fig. 4:**
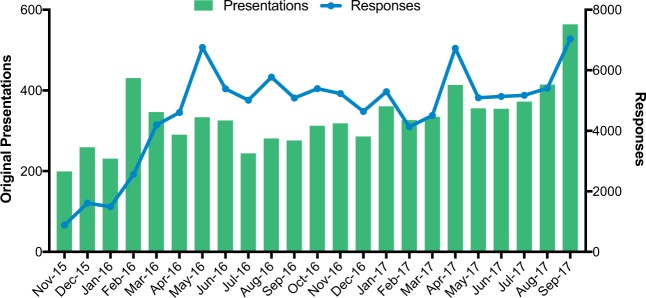
Monthly engagement on the platform. Number of original presentations (green bars, left *y* axis) and responses (blue line, right *y* axis) made each calendar month

Monthly engagement on the platform increased during the time period studied (Fig. 4). Linear regression analysis, excluding incomplete months, illustrated statistically significant increases in original presentations over time (slope 1.34, Pearson *r* = 0.5909, *p*-value 0.004) and responses (slope 30.48, Pearson *r* = 0.6053, *p*-value 0.003). We found that while the number of presentations made remained relatively consistent throughout the week with little variation, response activity was the highest on Sunday.

## Discussion

Smartphones and computers are pervasive in the outpatient and hospital environment, setting up the potential for virtual medical crowdsourcing and moving from the classic second opinion to the *n*^th^ opinion. Here, we aimed to understand this growing space by exploring the characteristics of early physician adopters and the usage statistics using a new digital medical app platform, Medscape Consult. We found rapid, voluntary engagement from physicians of all ages and medical sub-specialties from around the globe.

Medscape Consult is not the only platform for providing a real-time portal for virtual physician-to-physician engagement. Several crowdsourced diagnostic platforms have been evaluated though these studies have been small in size and from limited locations.^11,17,18^ Similar to Medscape Consult, many of the popular crowdsourced platforms are open solely to validated clinicians including the Human Diagnosis Project, Sermo, QuantiaMD and Fig. 1, the later having been touted as the “Instagram for doctors”, focusing on medical image sharing. Other platforms, such as HealthTap and CrowdMed, provide virtual dialogs between both patients and medical professionals in search for diagnostic answers. eConsult services within closed healthcare systems have been studied extensively. Although not considered crowdsourced platforms, when physicians were polled about the utility of these services, the eConsult services ranked highly for perceived improvement of patient management,^19^ providing value to patients and providers^20–22^ and the vast majority of participating physicians stated that they improved their medical knowledge.^23^ Collectively, digital online platforms aim to vastly expand resources and potentially provide for a more formalized and clear discussion of specific cases.

Here, we show that Medscape Consult was being utilized by physicians of all ages from around the world. Interestingly, there was an age offset for presenters and responders. Younger physicians were responsible for creating the majority of original presentations, with one-fifth created by physicians 26–30 years of age, while users who had presumably been in practice longer were engaged within this community with more responses. This could potentially be the result of better familiarity with social networking and mobile medical apps for younger physicians having trained in a digital era. However, with the majority (>60%) of all responses coming from physician users over 60 years of age, it is clear that older physicians feel comfortable with and support this type of virtual engagement. This certainly illustrates the broad appeal of a virtual medial consultation platform. The opportunity to query the Medcape Consult user base in addition to physicians on Medscape who are not using the Medscape Consult platform on their overall digital savvy and acceptance of crowdsourced decision-making tools such as this would be an appropriate next step to understand potential barriers. This would also be an important step in gaining insight on the age discrepancies related to virtual content creation in this cohort. The global reach of the platform was extraordinary with physician users from every continent creating original content as presentations or responses. As expected for a English language-based platform, the greatest number of users were registered in the United States and Europe, though users from Asia, Latin America, North Africa/Western Asia, and Central/Eastern Asia each made up at least 10% of the active user base on the platform. This global reach is increasingly important to physicians given the populations increased access to world travel and spread of diseases that traditionally had been limited geographically.

Given that physicians in Internal Medicine/General Practice and Family Medicine (included in the Internal Medicine classification here) make up the most populous specialty in practice, it was not surprising that physicians in this category were the highest user population and accounted for the highest percentage of engagement. Activity from Hematology/Oncology, especially the number of responses to original presentations was higher than their representation as active users. Conversely, engagement activity by Obstetrics/Gynecology was less than would be expected by the number of users on the platform.

Our study had several limitations. Most importantly, we do not have the final diagnoses for each presenter to determine whether peer input improved accuracy. This information would be vital to connecting improved diagnostic abilities using a crowdsourced virtual consultation platform and reducing diagnostic medical errors. A follow-up survey sent to users creating original presentations 1 week after going live may be a potential option for the platform to gain this data. Similarly for eConsult services, data pertaining to patient outcomes has been lacking and poorly reported in prior studies.^15^ The true reach of the platform is unknown as our analysis did not account for physicians who had access to the platform but did not engage in content creation. While there was overall growth in the creation of content during the study time, the proportion of users who only engaged once (through either a presentation or response) remain the majority. This remains an important concept for the success of platforms like this moving forward though outside the scope of this study. The patient–doctor experience is a personal one and that these “*n*^th^ opinions” took place virtually and only based on potentially biased information provided by the presenter, the responders are not afforded the opportunity to fully examine the patient. Thus, important aspects of the physical exam, interpersonal discussion, and subtle clues to diagnosis are likely lost in this digital space, which could potentially increase the risk of diagnostic inaccuracy. Similarly, aspects related to user experience and user interface that may impact long-term retention with digital tools remain unexplored but may be key factors in this lower than expected number of frequent users.

Artificial intelligence has been advocated as the definitive pathway for reducing misdiagnosis. But our findings suggest the potential for collective human intelligence, which is algorithm-free and performed rapidly on a voluntary basis, to emerge as a competitive or complementary strategy.

While there are certainly more refinements and study of this platform required, we have demonstrated an extraordinary reach and potential for a multispecialty, crowdsourced, global virtual consultation platform at scale for physicians in search of diagnostic input.

## Methods

### Sample

Participants were registered users of Medscape Consult (https://www.medscape.com/consult), a no-cost, online community where physicians are able to ask and answer clinical questions and discuss clinical challenges. Access to the online community was available via home computers, web-enabled mobile devices, and a mobile application. The study sample consisted of 37,706 physicians who created content between 10 November 2015 and 12 October 2017. During the time that this platform was studied, users were restricted to individuals who had received a medical degree. Content creation was defined as publishing an original presentation or response.

All participants completed an online account setup process and agreed to terms of service that allowed for data to be analyzed in aggregate. The study was conducted in accordance with all relevant guidelines and procedures and approved by the Scripps IRB.

### Statistical analysis

Demographic characteristics of the study sample were described according to categories of presenters (defined as those who published an original query or case) and responders (defined as those who only ever commented on an existing presentation) using univariate descriptive statistics (i.e., proportions and means and standard deviations). Univariate descriptive statistics were similarly used to describe the characteristics of the presentations and responses over time (months and day of the week) and according to medical specialty, geographic region of origin, device of origin, and categories of presenters and responders. Linear regression was used to examine change in monthly engagement. Original presentations were described by assessing the quantity and the amount of elapsed time between an original presentation and the first and last corresponding response was assessed using univariate descriptive statistics. All statistical analyses were conducted using STATA 13.0 (StataCorp, College Station, TX, USA).

## Electronic supplementary material

Supplementary Information

## Data Availability

No unique coding or algorithms were utilized for the analysis. De-identified data access will be considered as needed for other researchers.

## Electronic supplementary material

**Supplementary information** accompanies the paper on the *npj Digital Medicine* website (10.1038/s41746-018-0064-y).
