# Supplementary material for: From second to hundredth opinion in medicine: A global consultation platform for physicians
Source: NPJ Digit Med. 2018 Oct 9;1:55. doi: 10.1038/s41746-018-0064-y (PMC6550165; doi:10.1038/s41746-018-0064-y)
Supplement: Supplementary file 1 — Supplementary Information [file 41746_2018_64_MOESM1_ESM.pdf]

Example of original presentations and responses.

Editorial note: The text has been left unaltered from original post and may contain spelling errors.

EXAMPLE 1:

Original Presentation:
08/10/17
Hematemesis in an elderly sailor
A 65 year old sailor is admitted to hospital with the complaint of sudden vomiting of coffee-ground vomitus. He has had attacks of upper abdominal fullness following meals. On examination, he had a temp of 101 F, pulse 110, bp 100/70, anemic, not icteric. Chest clear. Liver palpable 2 fingers below costal margins, it's hard, nodular. Spleen enlarged. No ascites, no ecchymosis or petechia. Investigations show Hb 8.6 g/dl TLC 2000/mm3 plys 65% lymphos 33% myelocyte present band cells 2%. Platelets 40000/mm3. PT 21 seconds control 16 seconds. RBCs 2.5 million/mm3. Please discuss possible diagnosis

Responses:

Responding Physician #1
08/10/17
Hepatic etiology likely. Icteric? Unlikely variceal bleed, but clearly bleeding source below the ligament of Treitz

Responding Physician #2
08/10/17
GI bleeding perform gastrodeudonoscopy , bone marrow biopsy

Responding Physician #3
08/11/17
MDS
myelofibrosis
Lymphoma

Responding Physician #4
08/12/17
Variceal bleeding
Bleeding ulcer in hepatic patient

EXAMPLE 2:

Original Presentation:
03/15/17
Is this neurocysticercosis of the brain?
A 47-year-old man presented to my clinic on 13/03/2017 with a complaint of frequent seizures. Most of the seizures are focal motor clonic involving the left upper limb & left side of face. He has experienced these seizures since December, 2015, on an average of 2-3 times/day. On 3-4 occasions they became secondarily generalised. He is on antitubercular therapy(rifampicin, INH [isoniazid], ethambutol, & pyrazinamide) and levetiracetam 2000/day since December 2015. His serial brain MRI shows an enhancing granulomatous lesion in the right parietal cortex. Over this period of 16 months of anti-TB therapy, no change was noted in the lesion. His comorbidities include overweight (BMI 28.3), hypothyroidism (adequately replaced), diabetes mellitus controlled on 1700mg/day of metformin (last HbA1c of 5.8%). Our region of residence is India. My diagnosis now is neurocysticercosis. I plan to stop ATT, start him on zonisamide or topiramate, taper off levetiracetam, start a course of albendazole, continue metformin & follow him by another MRI brain 3 months later. Meanwhile, I will send for cysticercal serology. His serial brain MRI and 1st CT are attached. *[author's note: images excluded here]*

Responses:

Responding Physician #1
03/15/17
It could be. The 3rd picture seems to show a scolex within the cystic lesion. You could also check for cysticercin antibodies and maybe get some plain radiographs of the lower extremities to see if you can identify any intramuscular calcified cysts.

> *Post Author*
> *03/15/17*
> *Thanks. Clinically, I searched for subcutaneous nodules, I could locate none. I agree that in T2 sequence there is a possible scolex.*

Responding Physician #2
03/15/17
Is this patient HIV positive ? Is this a primary tuberculosis or extrapulmonary ?

> *Post Author*
> *03/15/17*
> *not tested as yet for HIV. No clinicoradiological evidence of pulmonary TB.*

> *Responding Physician #2*
> *03/15/17*
> *Don't stop antituberculosis therapy. It could be a tuberculoma. View the dilemma in diagnosis: www.jacpjournal.org/article.asp Treatment: www.ncbi.nlm.nih.gov/.../*

Post Author
03/15/17
So much thankful to you for sharing this enlightenening link. But is the ATT for more than one year not enough to clear the lesion? Only one scenario in which tubercular lesion would not be cleared is resistant TB. But then in that case it should have spread or increased in size. Is the scolex, seen in the picture 3, not diagnostic of NCC?(provided we agree that it is scolex)

Responding Physician #2
03/16/17
I saw the images 1 and 2 of december 2015. Those images seem related to Tuberculosis. The last image (2016 june) there is a black circle and an small image suggesting vesicular stage of the cysticercus (?). I agree to you that one year of treatment without results can be considered TB resistance or other diagnosis, but also co-infection. Therefore shall be realized cysticercal serology and also rule out HIV infection, TBM (Cytology and Microbiology), Toxoplamosis and Cryptococosis. Have you previous exams confirming TBM suspiction before of ATT ? Is the patient vegetarian and from endemical region of NCC ?

Responding Physician #3
03/27/17
HIV testing must be urgent!

Responding Physician #4
03/15/17
Agree with your diagnosis and management plan.
I have two concerns:
1. Did you perform a fundoscopic exam ?
2. I would consider steroids with a taper upon initiation of albendazole:
www.ncbi.nlm.nih.gov/.../

Responding Physician #5
03/26/17
I would prefer biopsy and further serology test for toxoplasma,fungal culture as well blood culture and CBC with diff to R/O other infectious and malignant condition
at this point differentia diagnosis to narrow down on final diagnosis is rule for success in management in this patient

Responding Physician #6
03/26/17
Is he a smoker? What radiographic evaluation was made of the lungs? If possible a CT of chest, abdomen and pelvis to rule out neoplasm. This is involves the motor strip, and surgery could

leave some deficits. If seizures are controlled, I think it is reasonable to follow up with another MRI within 3 months, with the treatments you described.

Responding Physician #7
03/26/17
Agree with anti-cystercin Abs and lower extremity radiographs. Zonisamide does take awhile to taper up given the long half-life - was he maximized on levetiracetam which is why you want to switch, given his ongoing seizures? What prompted the commencement of the anti-tuberculin therapy?

Responding Physician #8
03/26/17
It could be a tuberculoma;cisticercos are usually present in the ventricular sistem! I would recommend removal of the lesion

Responding Physician #9
03/26/17
This is called a "granulomatous" lesion. No way of knowing unless wee look at it under the scope

Responding Physician #10
03/26/17
This lesion should be surgically removed as it may be causing the patient's seizures. It may be a meningioma.

> *Post Author*
> *03/27/17*
> *I think obviously it is the cause of his seizures. Patient is refusing any invasive intervention.*

Responding Physician #11
03/26/17
TB lesion is possible and could be removed

> *Post Author*
> *03/27/17*
> *I think TB remains a highly unlikely possibility, since he has received 4-drug ATT for more than 16 months., with no improvement. Resistant TB remains an exception.*

Responding Physician #12
03/26/17
HIV status? Basis for original dx of TB? Regardless, because of the uncertainty of the etiology (TB, neurocysticercosis, tumor, other), the continued presence of seizures, and the peripheral, relatively accessible location of the lesion, I think that excision is the best course. In addition to being diagnostic, removal could be curative.

Post Author
03/27/17
HIV negative. I presume that original Dx of TB was based on clinical & imaging findings along with epidemiological high Prevalence of TB in our setting. Patient is refusing any invasive intervention.

Responding Physician #13
03/26/17
As a Neurosurgeon, I see this lesion as being near the surface, in an accessible location, causing trouble (seizures) and some surrounding edema, and therefore should be surgically removed. Patients with a history of TB should never be given steroids. I have seen patients who had TB 25 years earlier have it flare up just by being given "routine" post op steroids.

Responding Physician #14
03/27/17
I would suggest a PET, surgical removal and histological examination,
before any causal treatment

Responding Physician #15
03/27/17
I agree with surgical remove however becarful with motor area , you should make a good neuroimaging study before. And continue levetiracetam but increase dose to 3000 mg/day .

Responding Physician #16
03/27/17
I do agree with brain cysticercosis

Responding Physician #17
03/27/17
I agree with Dr. [X], with neuronavigation is a easy surgery and with the diagnosis you know what to treat

Responding Physician #18
03/27/17
Serology, leveritacetam Andrés albendazol

Responding Physician #19
03/27/17
I consider that it could be cysticercosis and that the cyst could be calcified. I would also check for antibodies and more images of the brain.

Responding Physician #20
03/27/17

Sifilis

Responding Physician #21
03/27/17
Looks more like a mets vs tuberculoma. I suggest you remove it

Responding Physician #22
03/27/17
An ring-enhancing granulomatous lesion is likely inflammatory, and in a diabetic with hypothyroidism, probably infectious, possibly an abscess. I don't see the scolex of neurocysticercosis nor hydrocephalus to justify abendazol, but will certainly start AED therapy with lacosamide or gabapentin, but not phenytoin due to interaction with INH!

> *Post Author*
> *03/27/17*
> *Does chronicity of disease not rule out Abscess?*

Post Author
03/27/17
What about Oxcarbamazepine.?

Responding Physician #23
03/28/17
Don't stop anti-tuberculosis, do biopsy for one of theses lesions, tuberculous lesion always not predictable, what about his Csf results , note : imaging finding of tuberculoma does not benefit in prognosis and follow up ,the patient clinical finding may resolve and tuberculous still present so it's not benefit in follow up or change in plan of management .

Responding Physician #24
03/30/17
I would prefer surgery. This is a superficial lesion and the approach is not difficult. The diferential of a tumor would be clarified. My first diagnosis is not neurocysticercosis due the long evolution time.

Responding Physician #25
03/30/17
Granulomatous lesion of neurocysticercosis

Responding Physician #26
03/30/17
Therapy of seizures of neurocysticercosis: phenytoin, dexamethazone or dexchorpheniramine maleate

Post Author

05/15/17
I have asked the patient to repeat MRI brain after few months. I will share the follow up. Meanwhile his seizures are controlled well with oxcarbazepine administration. I also administered a course of Albendazole empirically.

Post Author
05/20/17
Cysticercus serology negative.

Responding Physician #27
Jun 08
what would be sacrificed with neurosurgery . What is his probability of survival with removal of lesion. What did fudiscopy show. Any spasm ? Does he have other comorbidities . Thank you for sharing your case. it is like the old physicians on line.
